# Human inborn errors of the phagocyte respiratory burst: Chronic granulomatous disease and beyond

**DOI:** 10.70962/jhi.20260106

**Published:** 2026-09-18

**Authors:** Anna-Lena Neehus, Vijay G. Sankaran, Jean-Laurent Casanova, Jacinta Bustamante

**Affiliations:** 1 Division of Hematology & Oncology, Boston Children’s Hospital, Harvard Medical School, Boston, MA, USA; 2 Department of Pediatric Oncology, Dana-Farber Cancer Institute, Harvard Medical School, Boston, MA, USA; 3 Broad Institute of MIT and Harvard, Cambridge, MA, USA; 4 Howard Hughes Medical Institute, Boston, MA, USA; 5 Harvard Stem Cell Institute, Cambridge, MA, USA; 6 Laboratory of Human Genetics of Infectious Diseases, University of Texas Southwestern Medical Center, Dallas, TX, USA; 7 Center for the Genetics of Host Defense, University of Texas Southwestern Medical Center, Dallas, TX, USA; 8 Howard Hughes Medical Institute, Dallas, TX, USA; 9 Department of Immunology, University of Texas Southwestern Medical Center, Dallas, TX, USA; 10 Children’s Medical Center Research Institute, University of Texas Southwestern Medical Center, Dallas, TX, USA; 11 Department of Pediatrics, Necker Hospital for Sick Children, Assistance Publique-Hôpitaux de Paris, Paris, France; 12 Laboratory of Human Genetics of Infectious Diseases, Necker Branch, INSERM UMR 1163, Institut Imagine, Université Paris Cité, Paris, France; 13 Study Center for Primary Immunodeficiencies, Necker Hospital for Sick Children, Assistance Publique-Hôpitaux de Paris, Paris, France

## Abstract

The phagocyte respiratory burst is an effector mechanism of immunity by which granulocytes, monocytes, and other myeloid cells generate reactive oxygen species (ROS). Inborn errors of core phagocyte NADPH oxidase components (gp91^*phox*^, p22^*phox*^, p47^*phox*^, and p67^*phox*^) underlie most cases of chronic granulomatous disease, characterized by severe, recurrent bacterial and fungal infections. An expanding spectrum of inborn errors of immunity (IEIs) has been identified that affect molecules that trigger (IFN-γ, TNF, and their signaling pathways) or control NADPH oxidase assembly and activation (EROS, p40^*phox*^, PKCδ, RAC2, and DOCK2), thereby impairing ROS production. Studies of these genetic disorders, which include infection, autoinflammation, and autoimmunity, have identified crucial pathways governing the induction, regulation, assembly, priming, activation, and function of the phagocyte NADPH oxidase complex. We review here the diverse IEIs affecting phagocyte ROS production, highlighting disorders in which ROS generation is absent, reduced, or subset-specific, and the corresponding cellular, immunological, and clinical phenotypes.

## Teaser

This review covers the human genetic defects affecting the phagocytic respiratory burst, highlighting the dichotomy between defects of the core NADPH oxidase and of upstream regulators, and explores how these direct and indirect disruptions underlie infection, autoinflammation, and autoimmunity.

## The phagocyte nicotinamide adenine dinucleotide phosphate (NADPH) oxidase complex

The NADPH oxidases are a conserved family of transmembrane protein complexes that generate superoxide anions (O_2_^•–^) and downstream ROS by transferring electrons across biological membranes (1). In mammals, seven isoforms (NOX1–5, DUOX1, and DUOX2) have been identified, with roles ranging from host defense (NOX2) to intestinal (NOX1, DUOX2) or epithelial (DUOX1) homeostasis (2, 3). All NADPH oxidases have key structural features in common: a C-terminal NADPH-binding site, a flavin adenine dinucleotide (FAD)–binding region, six conserved transmembrane domains, and four essential heme-binding histidine residues (2). One member of this family, NOX2, also known as gp91^*phox*^, serves as the canonical phagocyte NADPH oxidase. Much of what we understand about its biology derives from the study of patients with chronic granulomatous disease (CGD) in the 1950s and 1960s (4, 5, 6). CGD was first described clinically in 1957 and was initially thought to be exclusively X-linked recessive (XR) (5). In 1966, it was demonstrated that neutrophils of CGD patients had normal phagocytic capacity but were unable to kill ingested bacteria (7). Subsequent publications directly linked this defect to an inability to generate O_2_^•–^ during the respiratory burst (4, 8). The recognition of affected female patients in 1968 demonstrated the existence of autosomal recessive (AR) forms of CGD (6). The nitroblue tetrazolium (NBT) test provided the first rapid functional screening assay for phagocyte oxidase activity (9). NBT testing has since largely been replaced by the more sensitive dihydrorhodamine (DHR) assay or other fluorescent probes (10, 11).

The absence of cytochrome *b* in CGD and its role in generating ROS during the phagocyte respiratory burst were first described in 1978 (12). The subsequent cloning of *CYBB* in 1986 identified the gene encoding gp91^*phox*^, confirming the genetic locus underlying XR CGD (13, 14). The gp91^*phox*^ subunit is the only catalytic component of the NADPH oxidase complex. It contains NADPH- and FAD-binding sites and two nonidentical heme groups, which mediate electron transfer from cytosolic NADPH to molecular oxygen (O_2_). In 1987, the transmembrane protein p22^*phox*^ (*CYBA*) was identified as the membrane subunit associated with gp91^*phox*^, forming the flavocytochrome b558 heterodimer (15, 16). Studies in cell-free systems showed that the cytosolic subunits p47^*phox*^ and p67^*phox*^, encoded by *NCF1* and *NCF2*, respectively, together with the small GTPase RAC2, were essential for activation of the phagocyte NADPH oxidase (17, 18, 19). The third cytosolic subunit, p40^*phox*^ (*NCF4*), was discovered in 1993 as a binding partner of p67^*phox*^ (20). In 2017, the chaperone EROS (*CYBC1*) was shown to be indispensable for gp91^*phox*^ expression in humans and mice due to its role in stabilizing gp91^*phox*^ during its maturation and glycosylation (21). Cryo-electron microscopy has provided detailed structural insight into the phagocyte NADPH oxidase itself. Structures of the core gp91^*phox*^–p22^*phox*^ complex in its resting state revealed the arrangement of the transmembrane helices and heme-binding sites, which facilitate electron transfer (22). A subsequent structure of the activated complex showed how the p67^*phox*^–RAC1 complex reconfigures gp91^phox^ to stabilize NADPH binding and enable electron transfer (23). The structure of the EROS–gp91^*phox*^–p22^*phox*^ heterotrimer showed that EROS engages gp91^*phox*^ at contacts distinct from the p22^*phox*^ interface and that it prevents interaction with FAD and NADPH, therefore preventing spontaneous activation of gp91^*phox*^ (24).

**Figure 1.**
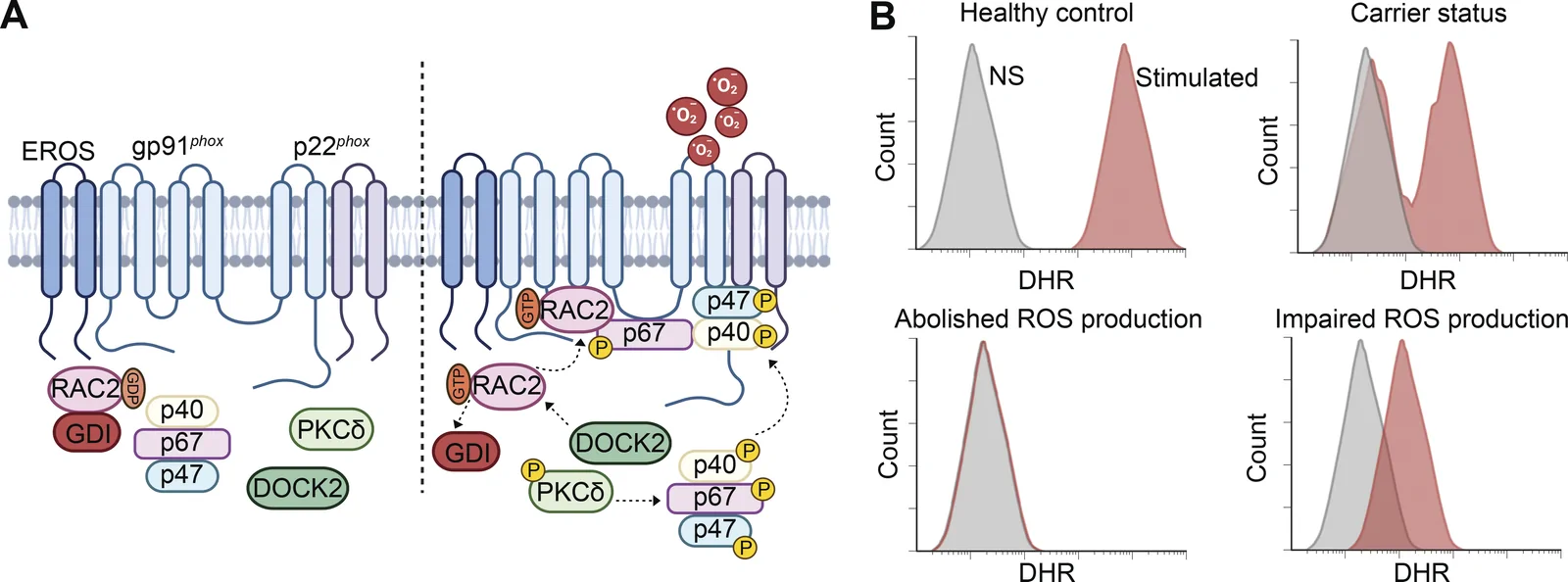
**NADPH oxidase assembly and the functional DHR assay. (A)** Schematic representation of the phagocyte NADPH oxidase complex in the resting (left) and activated (right) states. P, phosphorylation. **(B)** Schematic representation of DHR assay results for neutrophils showing a healthy control, a female carrier of a deleterious *CYBB* variant, and abolished and impaired DHR oxidation. NS, non-stimulated.

The phagocyte NADPH oxidase complex is activated by the binding of bacteria, fungi, or soluble inflammatory mediators to specific receptors, such as CD11b/CD18, which recognizes bacterial and fungal β-glucans and iC3b; the Fcɣ receptor, which binds IgG; and dectin-1, a receptor for β-glucans (1, 25). Receptor engagement triggers p47^*phox*^ phosphorylation by serine kinases, including members of the protein kinase C (PKC) family (Fig. 1 A). This phosphorylation induces changes in the conformation of p47^*phox*^, promoting translocation of the cytosolic p40^*phox*^/p47^*phox*^/p67^*phox*^ heterotrimer to the membrane, where p47^*phox*^ and p67^*phox*^ bind to p22^*phox*^ and gp91^*phox*^, respectively (1). Concomitantly, the small GTPase RAC2 is translocated to the membrane, where it dissociates from its GDP dissociation inhibitor (GDPI) and is converted to its active GTP-bound form by guanine nucleotide exchange factors (GEFs). RAC2-GTP then binds to the N-terminus of p67^*phox*^, inducing a change in conformation that facilitates the interaction of p67^*phox*^ with gp91^*phox*^ and its activation (26). Once fully assembled, the NADPH oxidase complex becomes catalytically active and generates O_2_^•–^ by transferring electrons from cytosolic NADPH to molecular O_2_ in the extracellular or phagosomal space. Within the phagosome, O_2_^•–^ is primarily dismutated to hydrogen peroxide (H_2_O_2_) by myeloperoxidase (MPO) rather than by spontaneous dismutation or superoxide dismutase (27, 28). MPO further catalyzes the reaction of H_2_O_2_ with halide (Cl^-^, Br^-^, and I^-^) or pseudohalide (SCN-) ions, generating hypohalous acids, including the highly cytotoxic and potent bactericidal hypochlorous acid (HOCl), which is the primary antimicrobial effector in neutrophil phagosomes (29). HOCl exerts its antimicrobial effects through rapid reactions with essential biological targets, such as lipids, nucleic acids, sulfur-containing amino acids, and other membrane components (30). In mouse models, it has also been shown that O_2_^•–^ production within the phagosomal lumen results in a compensatory K+ ion influx, thereby facilitating the release of cationic granule proteases such as elastase and cathepsin G from their proteoglycan matrix, which are potent antimicrobial effectors (31, 32).

Inborn errors affecting components of the phagocyte NADPH oxidase complex—p22^*phox*^, p40^*phox*^, p47^*phox*^, p67^*phox*^, gp91^*phox*^, and EROS—have been identified in humans (33, 34). These defects underlie CGD, the most frequent disorder of phagocyte ROS production, which has forms described as classic (complete) or variant (partial), depending on the degree of NADPH oxidase impairment. However, beyond CGD, an expanding spectrum of IEIs disrupting the expression, assembly, activation, or function of the phagocyte NADPH oxidase is increasingly being recognized. Here, we summarize the IEIs affecting the phagocyte respiratory burst, including disorders associated with absent, partial, or subset-specific ROS production, and highlight the molecular pathways governing NADPH oxidase expression and function. While inflammatory complications are a prominent feature of CGD and related disorders, this review focuses primarily on susceptibility to infection, with the inflammatory manifestations having been comprehensively reviewed elsewhere (35, 36, 37, 38, 39).

## Inborn errors of the phagocyte NADPH oxidase complex

### Hemizygous CYBB variants underlying CGD

Gp91^*phox*^, encoded by *CYBB* on the X chromosome, is the catalytic core of the phagocyte NADPH oxidase complex, essential for the generation of O_2_^•–^ during the phagocyte respiratory burst. XR gp91^*phox*^ deficiency is the most common genetic cause of CGD, accounting for ∼60–70% of cases. Hemizygous variants in *CYBB* have been reported in thousands of patients worldwide (40). These variants occur throughout the gene and include missense, nonsense, small out-of-frame insertions, deletions, duplications, indels, and splice-site variants (41). Regulatory variants have also been identified in the 5′ flanking region and introns. Large deletions encompassing *CYBB* and the neighboring genes on chromosome Xp21.1, also known as Xp21.1 continuous large deletion syndrome, can also cause a combination of XR CGD with Duchenne muscular dystrophy, the McLeod (Kell) phenotype, and XR retinitis pigmentosa. Patients with such deletions present with both features of CGD and associated syndromic manifestations (42). The study of patients with large Xp21 deletions was instrumental in mapping this genomic region and identifying *CYBB* as the genetic cause of XR CGD (43). Most pathogenic alleles result in an absence or very low levels of gp91^*phox*^ expression and/or complete loss of NADPH oxidase activity, although hypomorphic variants retaining residual O_2_^•–^ production have also been described (44). XR CGD is classified as X91°, X91^−^, or X91^+^ according to whether gp91^*phox*^ is undetectable or present at low or normal levels, respectively (40).

Clinically, CGD patients with XR gp91^*phox*^ deficiency present with severe, recurrent, often life-threatening bacterial and fungal infections due to *Staphylococcus aureus*, *Serratia marcescens*, *Burkholderia cepacia*, *Nocardia* species, and *Aspergillus* spp. (34) (Table 1). These infections may affect the lungs, brain, bone, skin, liver, spleen, and lymph nodes. Bacteremia and abscesses due to *Salmonella* have been reported in multiple CGD cohorts (45). CGD patients may experience adverse effects of Bacillus Calmette-Guérin (BCG) vaccination, ranging from regional disease (BCG-itis) to disseminated disease (BCG-osis) (39, 46, 47, 48, 49, 50). CGD patients are particularly susceptible to diverse forms of tuberculosis (TB) (46, 50, 51, 52, 53, 54). Disease due to environmental mycobacteria or parasites, such as *Leishmania*, has been reported in a few cases (46, 55). CGD patients often display inflammatory and granulomatous complications affecting the lungs, skin, gastrointestinal tract, retina, and genitourinary system (37, 38, 39, 56, 57). Most patients are diagnosed in infancy or childhood, and rarely during adulthood (58, 59, 60). Hypomorphic *CYBB* variants result in milder clinical phenotypes, late diagnosis, and better survival. Female carriers of pathogenic *CYBB* variants have a high prevalence of autoimmune manifestations (61). Unlike infectious complications, which are linked to skewed X inactivation of the wild-type allele, these clinical manifestations appear to be associated with the carrier state itself, although further studies are required to define the underlying mechanisms (61).

**Table 1.** Clinical manifestations of human inborn errors of the phagocyte respiratory burst

​	Gene (protein)	Inheritance	Defect	Clinical manifestations		
				Infection	Autoinflammation	Autoimmunity
Inborn errors of the phagocytic NADPH oxidase complex	*CYBA* (p22^*phox*^)	AR	Complete	Bacteria, mycobacteria, and fungi	++	+
			Partial	Bacteria, mycobacteria, and fungi	+	+/−
	*CYBB* (gp91^*phox*^)	XR	Complete	Bacteria, mycobacteria, fungi, and parasites	++	+
			Partial	Bacteria and fungi	+	+
			Partial^a^	Mycobacteria	Not reported	Not reported
	*NCF1* (p47^*phox*^)	AR	Complete	Bacteria, mycobacteria, and fungi	++	+
			Partial	Bacteria, mycobacteria, and fungi	++	+/−
	*NCF2* (p67^*phox*^)	AR	Complete	Bacteria, mycobacteria, and fungi	++	+
			Partial	Bacteria, mycobacteria, and fungi	++	+/−
Regulators of NADPH assembly, activation, and stability	*NCF4* (p40^*phox*^)	AR	Complete	Bacteria and mycobacteria	+++	+
	*PRKCD* (PKCδ)	AR	Complete	Bacteria, mycobacteria, fungi, and viruses	++	+++
	*CYBC1* (EROS)	AR	Complete	Bacteria, mycobacteria, fungi, and viruses	++	+
	*RAC2* (RAC2)	AD (DN)	Partial	Bacteria	Not reported	Not reported
		AD (GOF)	Partial	Bacteria and viruses	Not reported	Not reported
	*DOCK2*(DOCK2)	AR	Complete	Bacteria and viruses	Not reported	Not reported
Transcriptional and posttranslational regulators of NADPH oxidase	*TNF* (TNF)	AR	Complete	*M. tuberculosis*	Not reported	Not reported
	*IKBKG* (NEMO)	XR	Partial	Bacteria, mycobacteria, fungi, and parasites	+	Not reported
	*IRAK4* (IRAK-4)	AR	Complete	Bacteria	+	Not reported
	*MYD88* (MyD88)	AR	Complete	Bacteria	+	Not reported
	*NFKBIA* (IκBα)	AD (GOF)	Complete	Bacteria, mycobacteria, fungi, and parasites	Not reported	Not reported
	*IFNGR1* (IFN-γR1)	AR	Complete	Mycobacteria and intracellular microbes	Not reported	Not reported
	*IFNGR2* (IFN-γR2)	AR	Complete	Mycobacteria and intracellular microbes	Not reported	Not reported
	*IRF1* (IRF1)	AR	Complete	Mycobacteria and intracellular microbes	Not reported	Not reported
	*STAT1* (STAT)	AR	Complete	Mycobacteria and viruses	Not reported	Not reported

AD, autosomal dominant; AR, autosomal recessive; DN, dominant negative; GOF, gain-of-function; XR, X-linked recessive.

a Germline variants conferring MSMD.

XR complete gp91^*phox*^ deficiency results in abolished ROS production by all phagocytes, including neutrophils, eosinophils, monocytes, monocyte-derived macrophages (MDMs) and monocyte-derived dendritic cells (MDDCs), and EBV-transformed B (EBV-B) cells. The DHR assay is the most widely used test for CGD diagnosis, measuring the MPO- and H_2_O_2_-dependent oxidation of DHR to the fluorescent compound rhodamine 123 (Fig. 1 B and Table 2) (62, 63, 64). Critically, because DHR oxidation depends on both H_2_O_2_ generation by the NADPH oxidase and MPO activity, an abnormal DHR result is not specific for CGD (65). Complete MPO deficiency can also cause a strongly decreased DHR signal, mimicking CGD despite intact NADPH oxidase function, and has been reported as a cause of misdiagnosis on multiple occasions. Other assays, including ferricytochrome-*c* reduction, measure O_2_^•–^ directly and are not confounded by MPO activity (66, 67). They can help distinguish true CGD from MPO deficiency when an abnormal DHR result is obtained (63, 67). Functional studies have shown that loss of gp91^*phox*^ abolishes electron transfer from NADPH to molecular O_2_, thereby preventing the generation of O_2_^•–^ and downstream ROS. Defective NADPH oxidase activity may also impair neutrophil apoptosis and efferocytosis, contributing to the excessive inflammation and tissue injury observed in CGD (68). By contrast, certain *CYBB* variants may allow low levels of ROS production, correlated with later disease onset, reduced infectious burden, and higher overall survival (69). Standard treatment includes lifelong antibacterial and antifungal prophylaxis (70). IFN-γ is used in some cases (71, 72). Surgical intervention is indicated for osteomyelitis or invasive infections with a prolonged response to antimicrobial treatment. Corticosteroids are frequently used to treat colitis, often combined with other immunosuppressive agents, such as azathioprine or 6-mercaptopurine. Monoclonal antibodies targeting pro-inflammatory cytokines (anakinra, infliximab, adalimumab, and ustekinumab) have also been used to control autoinflammation and immune dysregulation (73, 74, 75, 76, 77). Allogeneic hematopoietic stem cell transplantation (HSCT) is curative and increasingly widely used, particularly in patients with severe disease or poor infection control (78, 79, 80). Lentiviral gene therapy has been employed and appears promising for treating XR CGD (81, 82).

**Table 2.** DHR results for neutrophils with inborn errors of immunity affecting NADPH oxidase activity

​	Gene (Protein)	Inheritance	Defect	DHR assay		
				PMA	*E. coli*/LPS	fMLF
Inborn errors of the phagocytic NADPH oxidase complex	*CYBA* (p22^*phox*^)	AR	Complete	Abolished	Abolished	Abolished
			Partial	Impaired	Abolished	Abolished
	*CYBB* (gp91^*phox*^)	XR	Complete	Abolished	Abolished	Abolished
			Partial	Impaired	Abolished	Abolished
			Partial^a^	Normal	Normal	Normal
	*NCF1* (p47^*phox*^)	AR	Complete	Abolished	Abolished	Abolished
			Partial	Impaired	Abolished	Abolished
	*NCF2* (p67^*phox*^)	AR	Complete	Abolished	Abolished	Abolished
			Partial	Impaired	Abolished	Abolished
Regulators of NADPH assembly, activation, and stability	*NCF4* (p40^*phox*^)	AR	Complete	Normal to impaired	Abolished	Abolished
	*PRKCD* (PKCδ)	AR	Complete	Impaired	Impaired	Not tested
	*CYBC1* (EROS)	AR	Complete	Impaired	Impaired	Not tested
	*RAC2* (RAC2)	AD (DN)	Partial	Normal	Not tested	Impaired
		AD (GOF)	Partial	Normal	Not tested	Increased
	*DOCK2* (DOCK2)	AR	Complete	Normal	Not tested	Not tested
Transcriptional and posttranslational regulators of NADPH oxidase	*TNF* (TNF)	AR	Complete	Normal	Normal	Not tested
	*IKBKG* (NEMO)	XR	Partial	Normal	Impaired	Impaired
	*IRAK4* (IRAK-4)	AR	Complete	Normal	Impaired	Impaired
	*MYD88* (MyD88)	AR	Complete	Normal	Impaired	Not tested
	*NFKBIA* (IκBα)	AD (GOF)	Complete	Not tested	Not tested	Not tested
	*IFNGR1* (IFN-γR1)	AR	Complete	Normal	Not tested	Not tested
	*IFNGR2* (IFN-γR2)	AR	Complete	Normal	Not tested	Not tested
	*IRF1* (IRF1)	AR	Complete	Normal	Not tested	Not tested
	*STAT1* (STAT1)	AR	Complete	Normal	Not tested	Not tested

AD, autosomal dominant; AR, autosomal recessive; DN, dominant negative; fMLF, N-formylmethionine-leucyl-phenylalanine; GOF, gain-of-function; XR, X-linked recessive.

a Germline variants affecting ROS production specifically in macrophages.

### Hemizygous CYBB variants underlying Mendelian susceptibility to mycobacterial disease

Germline hemizygous variants in *CYBB* are responsible for the most common form of CGD, in which NADPH oxidase activity is abolished or impaired. However, three hemizygous missense variants in *CYBB*—p.Q231P, p.T178P, and p.S333Y—have been described as responsible for Mendelian susceptibility to mycobacterial disease (MSMD) (83, 84). These patients developed infectious diseases due to *Mycobacterium tuberculosis*, *Mycobacterium bovis*-BCG, or *Mycobacterium avium* in the absence of other clinical manifestations characteristic of CGD (83) (Table 1). Functional testing in PLB985 cells showed that the p.Q231P and p.T178P variants were hypomorphic. When PLB985 cells were transduced with these variants and differentiated into macrophages, these cells failed to produce ROS, whereas ROS production was preserved in PLB985-derived neutrophils or monocytes. These three missense variants affected the NADPH oxidase activity of patient-derived MDMs and EBV-B cells (Table 2) (83, 84). Other phagocytes, including neutrophils, monocytes, and MDDCs, had a conserved respiratory burst even in the presence of physiological stimuli, such as *Escherichia coli* (83). The impact on ROS production correlates with gp91^*phox*^ protein levels across phagocyte types. Neutrophils had normal gp91^*phox*^ expression levels, whereas the patients’ MDMs and EBV-B cells displayed abnormally low levels. These cell type–specific differences in gp91^*phox*^ abundance may reflect distinct protein degradation pathways operating across phagocyte populations, as previously shown for some missense variants affecting p22^*phox*^ (85, 86). *CYBB* expression is regulated partly by IFN-γ, and efficient ROS production is crucial for the control of mycobacteria by tissue macrophages. Differences in ROS production between phagocyte subsets may, therefore, underlie the selective predisposition to mycobacteria. Treatment for these patients consists of antimycobacterial drugs and IFN-γ therapy.

### AR p22^phox^ deficiency

The membrane-bound subunit p22^*phox*^ (*CYBA*) heterodimerizes with gp91^*phox*^ to form flavocytochrome b_558_, the core scaffold required for NADPH oxidase stability and activity. The heterodimer formation in the ER is prerequisite for maturation and export to the plasma membrane. Unpaired gp91^*phox*^ or p22^*phox*^ is retained in the ER and degraded by the proteasome rather than trafficked further. Consequently, a null variant in either protein leads to loss of expression of both proteins (87, 88). At the plasma membrane, p22^*phox*^ interacts with the SH3 domains of p47^*phox*^ and p40^*phox*^ (89). Biallelic loss-of-function (LOF) or hypomorphic *CYBA* variants cause AR CGD and account for about 7% of CGD cases worldwide (90). Pathogenic variants include nonsense, frameshift, splice-site, missense, and large deletion alleles (90, 91, 92). Most alleles cause loss of p22^*phox*^ expression, leading to secondary loss of gp91^*phox*^ and complete absence of NADPH oxidase activity. Patients with AR p22^*phox*^ deficiency present clinically with a classic CGD phenotype, characterized by recurrent severe bacterial and fungal infections and granulomatous inflammation (Table 1). Inflammatory complications involving the lungs, gastrointestinal tract, and genitourinary system are frequent, and disseminated disease following BCG vaccination has been reported. Complete p22^*phox*^ deficiency abolishes ROS production in all phagocytes and EBV-B cells (Table 2). Standard management consists of lifelong antibacterial and antifungal prophylaxis and IFN-γ therapy. HSCT is curative and has been successfully performed in patients with severe disease, with outcomes comparable with other genetic forms of CGD (69).

### AR p67phox deficiency

P67^*phox*^, encoded by *NCF2*, is one of the cytosolic regulatory subunits of the phagocyte NADPH oxidase complex. It is essential for enzyme activation. P67^*phox*^ binds to the proline-rich repeats of p47^*phox*^ via its C-terminal SH3 domain (93). Upon stimulation, p67^*phox*^ becomes phosphorylated and is translocated to the membrane-bound flavocytochrome b558 complex, where it binds to RAC2, promoting its interaction with the gp91^*phox*^ subunit to facilitate the transfer of electrons from NADPH to the FAD center of the catalytic subunit (94). Biallelic LOF variants in *NCF2* cause AR CGD and account for ∼7% of CGD cases worldwide (90, 95, 96). Like patients with complete defects of gp91^*phox*^ or p22^*phox*^, patients with p67^*phox*^ deficiencies suffer from severe bacterial and fungal infections as well as inflammatory manifestations (Table 1). Patients often suffer from pneumonia, lymphadenitis, cutaneous and hepatic abscesses, osteomyelitis, and septicemia. ROS production is completely abolished in all phagocytes tested and in EBV-B cells from patients with complete p67^*phox*^ deficiency (Table 2). As in other forms of classic CGD, prophylactic treatment is effective, and HSCT is indicated in patients with severe disease (78, 80).

### AR p47phox deficiency

P47^*phox*^, encoded by *NCF1*, is a cytosolic organizer subunit that plays a key role in coordinating NADPH oxidase assembly. In resting cells, p47^*phox*^ is present in an autoinhibited conformation. Phosphorylation by protein kinases exposes the SH3 domains that bind to the proline-rich region of p22^*phox*^, thereby recruiting p67^*phox*^ to the membrane. *NCF1* has high homology with its two pseudogenes, *NCF1B* and *NCF1C*, with the same constitutive deletion (ΔGT) at the start of their presumptive exon 2 (97). Recombination events between *NCF1* and its pseudogenes are probably responsible for the acquisition of ΔGT variants at the resulting recombined *NCF1* locus (98). High-throughput sequencing has further mapped crossover breakpoints between *NCF1* and its pseudogenes (99, 100). AR p47^*phox*^ deficiency is the most common form of AR CGD, accounting for ∼20–25% of all CGD cases (90). Most patients (>80%) are homozygous for the ΔGT deletion at the start of exon 2 of *NCF1* (90, 92, 99, 101). Patients with Williams–Beuren syndrome, characterized by a 1.5-Mb deletion in 7q11.23, may also be diagnosed with p47^*phox*^ deficiency if their deletions include *NCF1* (102, 103, 104). Patients with AR p47^*phox*^ deficiency are susceptible to recurrent bacterial and fungal infections, including pneumonia, lymphadenitis, skin infections, and adverse reactions to the BCG vaccine (Table 1) (47). Inflammatory diseases, predominantly of the lungs and bowel, are also frequently observed (105). Genome-wide association studies have identified a common hypomorphic *NCF1* missense variant (rs201802880), shown to reduce ROS production, as a risk factor for various autoimmune diseases (106, 107). The level of ROS production by neutrophils is slightly higher in patients with complete p47^*phox*^ deficiency than in neutrophils from patients with other subtypes of CGD, but it remains abnormally low (Fig. 1 B and Table 2). Allogeneic HSCT is indicated and has been shown to decrease disease burden effectively (105). Autologous CD34^+^ HSCT with prime editing to correct the frequent ΔGT variant has recently been shown to restore NADPH oxidase activity in early-stage clinical trials (108).

## Regulators of NADPH oxidase assembly, activation, and stability

NADPH oxidase activity is dependent on the precise assembly of cytosolic and membrane-bound components into a functional complex. This process is orchestrated by proteins that control subunit recruitment, activation, and stability. Defects in these regulators, including p40^*phox*^, PKCδ, EROS, RAC2, and DOCK2, compromise ROS production and give rise to distinctive cellular and clinical phenotypes.

### AR p40^phox^ deficiency

*NCF4* encodes p40^*phox*^, a protein with three domains: an N-terminal PX-binding domain, a central SH3 domain that binds p47^*phox*^, and a C-terminal PB1 domain that interacts with p67^*phox*^. Biallelic LOF or hypomorphic *NCF4* variants have been reported in the PX and SH3 domains and occur in homozygous or compound heterozygous states (109, 110, 111, 112, 113, 114). 24 patients had symptoms, whereas four remained asymptomatic throughout adolescence; all the patients reported to date are alive. Approximately half the patients develop cutaneous inflammation (discoid lupus, eczema, erythema nodosum, and pyoderma gangrenosum), and granulomatous gastrointestinal manifestations (oral ulcers, Crohn-like inflammatory bowel disease [IBD]) (Table 1) (109, 110). Immune dysregulation may include immune thrombocytopenia and pediatric systemic lupus erythematosus (SLE) (111, 113). Noninvasive infections were reported in 42% of patients and included cutaneous lesions or adenitis, mostly caused by bacteria, such as *S. aureus* or BCG. One patient had hepatic and splenic abscesses due to *Candida,* and another presented with disseminated histoplasmosis (110). P40^*phox*^-deficient neutrophils and monocytes had low to normal levels of DHR staining in tests performed after PMA stimulation, whereas ROS production was much lower in response to *S. aureus* or *E. coli* (Table 2) (110, 111, 115). In addition, p40^*phox*^-deficient neutrophils showed defective *S. aureus* killing (109, 110). ROS production was also impaired in the patients’ EBV-B cells (110). NADPH oxidase activity in MDMs and MDDCs from p40^*phox*^-deficient patients is normal in response to PMA and LPS (110, 111). These findings highlight the critical role of p40^*phox*^ in phagocytosis-induced NADPH oxidase activation due to its N-terminal PX domain that binds specifically to phosphatidylinositol 3-phosphate (PI(3)P), a phosphoinositide that accumulates on phagosomal membranes (109, 116). By contrast, the PX domain of p47^*phox*^ binds PI(3,4)P_2_ and PI(3,4,5)P_3_, which are enriched at the plasma membrane upon activation (117). PI(3)P binding by p40^*phox*^ has been shown to be largely dispensable for PMA-induced ROS production, which instead relies on p47^*phox*^-driven assembly at the plasma membrane (117). This distinction may explain the selective phagocytosis-induced ROS defect and the generally milder infectious phenotype of p40^*phox*^-deficient patients. (110). In contrast, autoinflammatory and autoimmune manifestations are common and frequently represent the initial clinical presentation (109, 110, 111). The mechanisms underlying autoimmunity in p40^*phox*^ deficiency remain unclear, though impaired ROS-dependent B cell functions in B cells have been proposed (118). Treatments for p40^*phox*^ deficiency include antibiotics and corticosteroids, TNF biologics, and hydroxychloroquine to resolve hyperinflammation. In severe cases, allogeneic HSCT may be considered as a curative treatment (110).

### AR PKCδ deficiency

*PRKCD* encodes PKCδ, a ubiquitously expressed cytosolic kinase that regulates apoptosis, proliferation, and cell survival. PKCδ interacts with and phosphorylates the cytosolic NADPH oxidase subunits p40^*phox*^ and p47^*phox*^ (115). Phosphorylation of the T154 residue of p40^*phox*^ is a key regulatory step, leading to assembly of the cytosolic NADPH oxidase subunits with the membrane-bound components (119). AR PKCδ deficiency, caused by biallelic LOF *PRKCD* variants, was initially described as a monogenic cause of early-onset SLE (Table 1). To date, 30 patients have been reported, most with autoimmunity, antinuclear antibodies, and lymphoproliferation (115, 120, 121, 122, 123, 124, 125, 126, 127, 128, 129, 130, 131, 132, 133). PKCδ-deficient patients also presented with fungal and bacterial infections, reminiscent of those observed in CGD (115). Infections can be severe and include gastroenteritis, otitis media, lymphadenitis, sepsis, meningitis, pneumonia, gingivitis, and disseminated *Candida* infection (115, 120, 121, 122, 123, 124, 125, 126, 127, 128, 129, 130, 131, 132, 133). Frequent pathogens include *Haemophilus influenzae*, *Pseudomonasaeruginosa*, *Salmonella*, and *Streptococcus pneumoniae* (115, 123, 124, 125, 133). One patient suffered from infection with *Legionella pneumonia*. *Achromobacter xylosoxidans* or *B*. *cepacia* was isolated from patients with recurrent upper respiratory tract infections and cervical lymphadenitis (122). Viral infections (EBV, CMV, varicella-zoster virus, HHV6, HHV7, and SARS-CoV-2), which are rare in classic CGD, have also been reported (115, 124, 126, 127, 130). Four patients died due to sepsis, whereas one 6-year-old patient remained asymptomatic (115, 120, 124). PKCδ deficiency impairs ROS production in all phagocytes and patient-derived EBV-B cells (Table 2) (115). Importantly, PKCδ deficiency results in abnormal DHR assay results for neutrophils and monocytes stimulated with PMA or *E. coli* and can thus resemble variant CGD (122). It is therefore important to test for PKCδ deficiency in young patients with CGD-like infectious phenotypes and low levels of DHR staining. Management includes corticosteroids, sometimes combined with anti-CD20 therapy (115). Additional immunosuppressive options include hydroxychloroquine, mycophenolate mofetil, azathioprine or sirolimus, and the more recently released leniolisib (123). Prophylactic intravenous immunoglobulin and antibiotics are recommended.

### EROS deficiency

The EROS protein is encoded by *CYBC1* (*C17orf62)*, which is highly expressed in neutrophils, monocytes, and macrophages (21). EROS is a molecular chaperone consisting of 187 amino acids with an N-terminal Pleckstrin homology domain, two transmembrane α-helices, and a C-terminal tail. It interacts directly with gp91^*phox*^ in early gp91^*phox*^ biogenesis, stabilizing the gp91^*phox*^ precursor in the ER and supporting formation of the gp91^*phox*^-p22^*phox*^ heterodimer (86). To date, 13 patients with biallelic LOF *CYBC1* variants have been reported (134, 135, 136, 137, 138, 139). These patients suffered from infectious diseases due to bacteria (*S. pneumoniae*, *Salmonella*, *Stenotrophomonas maltophilia*, and *B*. *cepacia*), including mycobacteria (BCG, *M. tuberculosis*), viruses, and fungi (*Cladophialophora* sp., *Malassezia restricta*) (Table 1). In addition, EROS-deficient patients developed sarcoidosis, Crohn’s disease, autoimmune hemolytic anemia, granulomatous colitis, and IBD. Knockout of *CYBC1* in PLB985 cells resulted in the abolition of gp91^*phox*^ expression and a major decrease in p22^*phox*^ expression (86). AR EROS deficiency results in impaired ROS production by neutrophils in response to PMA, *E. coli*, and zymosan (Table 2) (135, 138). Monocytes also display lower levels of ROS production in the DHR assay in response to PMA and *E. coli*, whereas EROS-deficient EBV-B cells display a complete abolition of ROS production (138). EROS loss results in low gp91^*phox*^ levels in neutrophils, monocytes, and EBV-B cells, with the strongest impact on gp91^*phox*^ expression observed in EBV-B cells (138). Residual gp91^*phox*^ expression and respiratory burst activity in neutrophils and monocytes likely explain the milder infectious phenotype in EROS deficiency. Treatment includes appropriate drugs for the prevention of infections and inflammatory complications. HSCT has been reported to be an effective curative treatment in two patients (140).

### RAC2-related immunodeficiency

The small GTPase RAC2 (*RAC2*) is expressed exclusively in hematopoietic cells, in which it acts as a molecular switch regulating multiple downstream processes. In its GTP-bound, active state, it mediates activation of the NADPH oxidase by binding to p67^*phox*^, but it also regulates other cellular processes, such as actin polymerization, intracellular rearrangements, and increases in intracellular Ca^2+^ concentration. Pathogenic variants in *RAC2* underlie a spectrum of clinical phenotypes correlated with RAC2 activity: heterozygosity for constitutively active variants underlies neonatal severe combined immunodeficiency (SCID), whereas heterozygosity for dominant-negative variants causes infantile neutrophilic disorder, resembling leukocyte adhesion deficiency (141, 142, 143, 144, 145). A milder clinical phenotype with later-onset combined immunodeficiency (CID) is observed in patients heterozygous for dominant activating variants or homozygous for null variants (146). Sepsis is commonly observed in patients with constitutively active disease and omphalitis; abscesses and bacterial infections are frequent in patients with dominant-negative variants. RAC2-deficient patients with CID frequently present with upper and lower respiratory tract infections, viral infections (HSV, HPV), bacterial infections, and malignancies (Table 1) (141).

As for the clinical heterogeneity in patients carrying pathogenic *RAC2* variants, the associated cellular phenotypes are shaped by the nature of the underlying pathogenic variant (Table 2). DHR testing on neutrophils from patients with dominant-negative *RAC2* variants showed an abolition or severe impairment of ROS production in response to formyl-Met-Leu-Phe peptide (fMLF), which signals through the G-protein–coupled receptor FPR1 and activates RAC2 (147). By contrast, ROS production in response to PMA, which stimulates the NADPH oxidase in a RAC2-independent manner, was found to be unaffected. Neutrophils from patients with dominant activating RAC2 variants also responded normally to PMA but displayed higher levels of O_2_^•–^ production in response to fMLF and other formyl peptide receptor agonists (146, 148). These cells also had high basal O_2_^•–^ levels (141). Patients with constitutively active *RAC2* variants present with SCID and lack circulating neutrophils. Overexpression of mutant RAC2 in HEK293T cells results in an increase in O_2_^•–^ production both at baseline and in response to PMA (149). These findings demonstrate that RAC2 variants can dysregulate NADPH oxidase activity, reflecting the diverse clinical phenotypes observed in RAC2 deficiency. Treatment depends on the underlying genetic disorder, but HSCT is the only curative.

### DOCK2 deficiency

DOCK2 acts upstream of RAC1 and RAC2 as a GEF, activating these small GTPases by exchanging GDP with GTP. DOCK2 is crucial for chemokine-induced lymphocyte migration, actin polymerization, T cell proliferation, natural killer (NK) cell degranulation, and NADPH oxidase activation (150). DOCK2 deficiency, due to biallelic LOF variants in *DOCK2*, was first described in five unrelated children with early-onset bacterial and viral infections, T cell lymphopenia, and defective T, B, and NK cell responses (Table 1) (150). The clinical phenotype of DOCK2-deficient patients may resemble that of patients with leaky SCID or Omenn syndrome. DOCK2 loss affects the phagocyte respiratory burst by impairing RAC2 activation, resulting in reduced NADPH oxidase activity. Neutrophils from a DOCK2-deficient patient displayed impaired ferricytochrome-*c* reduction in response to PMA, whereas the results of DHR assays in DOCK2-deficient neutrophils and monocytes were normal in response to PMA (Table 2). The patient’s EBV-B cells displayed very low levels of O_2_^^−^^ and H_2_O_2_ production in response to PMA (151). Studies using DOCK2-deficient THP-1 cells provided further support for a DOCK2-dependent mechanism of ROS production. ROS production in response to stimulation with Curdlan, Mannan, or HKCA was much weaker in DOCK2-deficient THP-1 cells than in wild-type cells (152). Importantly, this phenotype was rescued by lentiviral overexpression of wild-type DOCK2, directly linking DOCK2 loss and abnormally low levels of ROS production. Murine studies have provided additional support for this, with ROS production markedly reduced in *Dock2*^−/−^ neutrophils in response to fMLF and PMA, and double knockout of both *Dock2* and *Dock5* resulting in even more severe defects in RAC1/RAC2 activation and ROS production than DOCK2 deficiency alone (153, 154). Taken together, these data support a role for the DOCK2-RAC axis in the phagocyte respiratory burst and the notion that DOCK2 deficiency also affects human phagocyte function. The treatment of DOCK2-deficient patients focuses on the use of antibiotics, antiviral therapies, and immunoglobulins to reduce the risk of severe infections. The only curative option is HSCT.

## Transcriptional and posttranslational regulators of the NADPH oxidase

**Figure 2.**
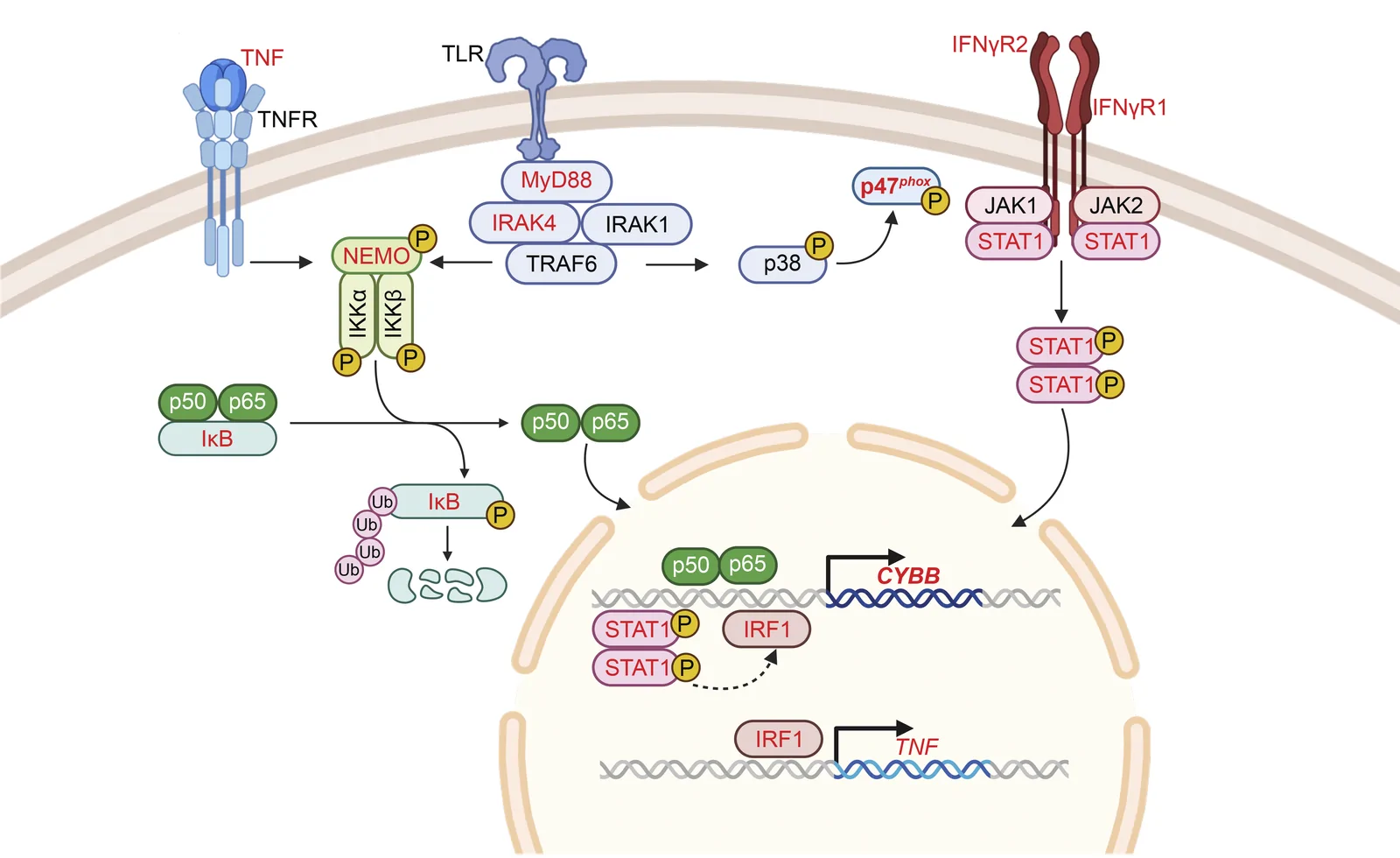
**Signaling pathways regulating the expression and activation of NADPH oxidase subunits.** Proteins in which genetic defects have been shown to affect NADPH oxidase expression/activation in humans are highlighted in red. NADPH oxidase core subunits are highlighted in bold red. P, phosphorylation; Ub, ubiquitination.

Upstream signaling pathways couple extracellular stimuli to NADPH oxidase activity by coordinating both transcriptional programs and posttranslational events (Fig. 2). Defects in these pathways typically present as broader immunological and clinical phenotypes, but they also compromise NADPH oxidase activity.

### Human TNF deficiency

TNF is a pro-inflammatory cytokine and key mediator of innate immune responses. TNF is produced primarily by myeloid cells, including monocytes and macrophages, in response to microbial stimuli and pro-inflammatory cytokines, such as IL-1β or IFN-γ (155, 156) (Fig. 2). In 2024, two related patients were reported to have complete TNF deficiency due to homozygosity for a private *TNF* frameshift variant (157). Both presented with recurrent pulmonary TB relapsing within 1 year of treatment but were otherwise healthy with normal inflammatory reactions throughout their lives (Table 1). One of these patients also suffered from listeriosis during pregnancy. Functional studies showed that TNF deficiency impaired ROS production in only a subset of macrophages, with no impairment detected in other ROS-producing cells (Table 2) (157). Respiratory burst impairment was observed specifically in GM-CSF–matured MDMs from both patients and in healthy control GM-CSF–matured MDMs and alveolar macrophage-like cells that differentiated in the presence of TNF-blocking antibodies in vitro. Likewise, treatment with TNF blockers of primary lung macrophages from healthy donors ex vivo also decreased ROS production. Mechanistic investigations in isogenic induced pluripotent stem cells (iPSCs) with a knockout of *TNF* and the genes encoding both its receptors, *TNFR1* and *TNFR2*, showed that only iPSC-derived GM-CSF–matured macrophages lacking TNF or TNFR1 displayed impaired ROS production after stimulation with PMA or heat-killed *M. tuberculosis*. This suggests TNFR1 signaling is essential for NADPH oxidase activation and respiratory burst-dependent antimycobacterial immunity in macrophages. Consistent with these findings, TNF signaling via TNFR1 has been shown to regulate NADPH oxidase activation directly by controlling the incorporation of FAD into NADPH oxidase enzymes (158, 159).

### Defects affecting canonical NF-κB activation

MyD88 is a key cytosolic adaptor molecule linking Toll-like receptors (TLRs) and IL-1 receptors to the IRAK complex, which consists of two active kinases (IRAK-1 and IRAK-4) and two noncatalytic subunits (IRAK-2 and IRAK-3) (Fig. 2). The activation of MyD88–IRAK-4-dependent pathways leads to activation of the MAPK and NF-κB pathways and production of pro-inflammatory cytokines. Canonical NF-κB activation is mediated by the NF-κB essential modulator (NEMO; *IKBKG*), which is essential for phosphorylation of the NF-κB inhibitor IκBα (*NFKBIA*), resulting in its degradation and the translocation of NF-κB dimers to the nucleus. Biallelic pathogenic variants in *MYD88* and *IRAK4* result in AR MyD88 and AR IRAK-4 deficiency, respectively. These two conditions have the same clinical phenotype: susceptibility to recurrent, invasive bacterial infections caused by pyogenic bacteria (Table 1) (160, 161, 162, 163). Monoallelic pathogenic variants in *IKBKG* (located on the X chromosome) confer susceptibility to infectious diseases in male patients. Affected patients display a narrow susceptibility to bacterial infections and only rare viral, parasitic, and fungal infections. Noninvasive infections commonly affect the upper respiratory tract and skin and are often caused by *P. aeruginosa* and *S. aureus*, respectively. Monoallelic gain-of-function (GOF) variants in *NFKBIA* cause an autosomal dominant disease with impaired NF-κB activation, often presenting as ectodermal dysplasia and broader susceptibility to infections, including those due to opportunistic pathogens (164, 165). The major clinical risk is invasive infections, mostly invasive pneumococcal disease, which occurs early in life and often recurs (161).

IRAK-4– or MyD88-deficient neutrophils display normal PMA-induced ROS production (Table 2) (166, 167). However, the stimulation of IRAK-4–deficient neutrophils with weaker agonists, such as LPS and fMLF, results in markedly lower O_2_^•–^ production (160, 168). Similar results have been obtained for cells from NEMO-deficient patients (168). Neutrophils from a patient with combined MyD88 and CARD9 deficiency displayed normal ROS production in response to PMA and *E. coli*, but monocytes showed reduced ROS production in response to *E. coli* (169). The loss of IRAK-4/MyD88 signaling impairs p38 MAPK activation, critical for p47^*phox*^ phosphorylation (170). Reduced p47^*phox*^ phosphorylation compromises NADPH oxidase activation and ROS production. Furthermore, EBV-B cells from patients with impaired canonical NF-κB signaling (LOF *IKBKG* or GOF *NFKBIA*) have abolished PMA-induced O_2_^•–^ production due to the lower *CYBB* expression resulting from the loss of p50 binding to its promoter (171). This phenotype was reproduced in U937 cells expressing the same variants (171). These findings highlight the role of MyD88–IRAK-4 and NF-κB signaling in coordinating innate immune responses to bacteria, including NADPH oxidase activation.

### Inborn errors of IFN-γ immunity

IFN-γ is the key cytokine involved in host defense against nonviral infectious agents, including intramacrophage bacteria (Fig. 2). Signaling via the IFN-γ receptor–associated kinases JAK1 and JAK2 leads to STAT1 phosphorylation, inducing the transcription of IFN-stimulated genes (ISGs), such as the gene encoding transcription factor IRF1, which can, in turn, induce the transcription of secondary response genes (172). Molecular defects of IFN-γ–mediated immunity underlie MSMD, a rare condition characterized by susceptibility to weakly virulent mycobacteria (Table 1). Genetic defects affecting IFN-γ responsiveness impair or abolish induction of ISGs encoding effectors of resistance to intracellular pathogens (173). IFN-γ stimulation promotes gp91^*phox*^ expression in human myeloid cells through the binding of the transcription factors STAT1, IRF1, and PU.1 to cis regulatory elements upstream of *CYBB* (174, 175, 176). Studies with patient-derived cells from patients with AR complete IFN-γR1 or IFN-γR2 deficiency have revealed a selective impairment of NADPH oxidase activity in MDMs and EBV-B cells (Table 2) (177). A similar cellular phenotype was reported in a patient with AR complete IRF1 deficiency or complete AR STAT1 deficiency, with low levels of ROS production in MDMs and iPSC-derived macrophages (156, 178). These data support a role for IFN-γ in regulating NADPH oxidase activity and suggest that impairment of the phagocyte respiratory burst contributes to mycobacterial susceptibility in patients with IEIs of IFN-γ–mediated immunity.

## Conclusions and perspectives

The study of human inborn errors of the phagocyte respiratory burst has provided fundamental insight into the molecular mechanisms governing ROS production and their essential role in host defense. Defects of core components of the NADPH oxidase complex define CGD and highlight the nonredundant requirement for phagocyte-derived ROS for protection against bacterial and fungal pathogens. Beyond these canonical forms, studies of the expanding spectrum of IEIs have revealed that partial, cell type–restricted or context-dependent impairments of ROS generation can give rise to diverse clinical and cellular phenotypes. Selective deficiencies of the respiratory burst in macrophages have provided direct evidence for a nonredundant role of macrophage-derived ROS in antimycobacterial immunity. Hemizygous hypomorphic variants in *CYBB* that selectively impair ROS production in macrophages cause MSMD, with infections restricted to *M. tuberculosis*, BCG, or environmental mycobacteria and relative resistance to pyogenic bacteria and fungi (83, 84). Similarly, patients with inherited TNF deficiency have a selective impairment of the respiratory burst in alveolar macrophages, whereas other phagocytic cell populations remain unaffected (157). The highly restricted infectious phenotype of recurrent pulmonary TB in these patients further highlights the crucial role of tissue-specific macrophage-dependent ROS production in host resistance to *M. tuberculosis.* The magnitude of residual NADPH oxidase activity shapes disease severity. Patients with hypomorphic variants underlying variant CGD retain partial ROS production and may present with milder or later-onset infections than those with classic CGD, indicating that even low levels of ROS can confer partial antimicrobial protection (69). Finally, defects in upstream NADPH oxidase regulators often cause broader phenotypes due to their pleiotropic functions. For example, PKCδ deficiency impairs not only NADPH oxidase activation but also NK cell function and B cell apoptosis, resulting in a broader clinical phenotype (115, 126). In these conditions, impaired ROS production probably contributes to, but does not fully account for, the infectious phenotype.

Human genetics has proved instrumental for elucidation of the regulatory mechanisms and signaling pathways of the NADPH oxidase that were not fully appreciated from experimental models alone. Hypomorphic variants and defects affecting only subsets of phagocytes underscore the need to consider cellular context, time, and type of stimulus when interpreting functional assays and clinical phenotypes. Neutropenia and certain genetic deficiencies, such as MPO or glucose-6-phosphate dehydrogenase deficiency, which have several clinical manifestations in common with CGD, can yield false-positive results in the DHR assay; careful interpretation of screening assays is therefore required, in conjunction with complementary functional and genetic analyses (34, 179). Standard functional assays of the phagocyte respiratory burst, including the DHR assay, typically employ PMA as a stimulus, which activates PKC directly and bypasses upstream signaling pathways, potentially missing more subtle, stimulus-dependent impairments in ROS production. A broader repertoire of physiological stimuli in functional testing would better capture the full spectrum of respiratory burst disorders. Improvements in genetic diagnosis, including broader access to next-generation sequencing and functional validation platforms, will continue to expand the catalog of known respiratory burst-associated disorders and will guide precision-medicine approaches and improve patient outcomes.
